# Unveiling the ups and downs of *miR-205* in physiology and cancer: transcriptional and post-transcriptional mechanisms

**DOI:** 10.1038/s41419-020-03192-4

**Published:** 2020-11-15

**Authors:** Elena Ferrari, Paolo Gandellini

**Affiliations:** 1 Department of Applied Research and Technological Development, Fondazione IRCCS Istituto Nazionale dei Tumori, 20133 Milan, Italy; 2 Department of Biosciences, University of Milan, 20133 Milan, Italy; 3 Present Address: Istituto Zooprofilattico Sperimentale della Lombardia e dell’Emilia Romagna, 25124 Brescia, Italy

**Keywords:** Cancer epigenetics, miRNAs

## Abstract

*miR-205* plays important roles in the physiology of epithelia by regulating a variety of pathways that govern differentiation and morphogenesis. Its aberrant expression is frequently found in human cancers, where it was reported to act either as tumor-suppressor or oncogene depending on the specific tumor context and target genes. *miR-205* expression and function in different cell types or processes are the result of the complex balance among transcription, processing and stability of the microRNA. In this review, we summarize the principal mechanisms that regulate *miR-205* expression at the transcriptional and post-transcriptional level, with particular focus on the transcriptional relationship with its host gene. Elucidating the mechanisms and factors regulating *miR-205* expression in different biological contexts represents a fundamental step for a better understanding of the contribution of such pivotal microRNA to epithelial cell function in physiology and disease, and for the development of modulation strategies for future application in cancer therapy.

## Facts

- *miR-205* contributes to the development of epithelia.
- *miR-205* is up- or down-modulated in different epithelial cancers.
- *miR-205* expression is regulated at the transcriptional and post-transcriptional level.
- *miR-205* biogenesis is intimately linked to the processing of its host gene (*MIR205HG*).
- *miR-205* and *MIR205HG*-derived long noncoding RNA play independent but complementary functions in epithelial cells.

## Open questions

- Which is the exact role of the sequences upstream or downstream of *miR-205*/*MIR205HG* locus?
- Do specific regions account for a *MIR205HG*-independent *miR-205* biogenesis?
- Which mechanisms or factors modify the reciprocal production of *miR-205* and *MIR205HG* long noncoding RNA?

## Introduction

MicroRNAs (miRNAs) are small, noncoding RNAs that play a critical role in a wide range of physiologic and pathologic processes, mainly by acting as negative post-transcriptional regulators of their target genes^1^. A tight control of miRNA expression is essential for tissue homeostasis and development. Indeed, several studies showed that dysregulated miRNA expression is functionally related to the onset of different human diseases, including cancer^2^.

Intergenic miRNAs are transcribed by RNA polymerase II or III, in the nucleus, as primary miRNA transcripts (pri-miRNA), which are then processed by Drosha-DGCR8 microprocessor complex into 70–90 bases long hairpin precursor miRNAs (pre-miRNA). Once exported to the cytoplasm, the pre-miRNA is cleaved by the RNAse III, Dicer, to produce the miRNA duplex, of which one strand is incorporated into the Argonaute complex to be guided to the target mRNA^3^. Alternative miRNA biogenesis pathways may occur for non-intergenic miRNAs, such as intronic ones, which are transcribed together with their “host genes” and then directly processed by the splicing machinery^4,5^. Therefore, the location of the miRNA transcription unit dictates different mechanisms of biogenesis.

MicroRNA-205 (*miR-205*) is a highly conserved miRNA expressed in epithelial tissues of different species^6,7^. In the human genome, the pre-*miR-205* sequence is located in the last intron/exon junction of a gene annotated as *miR-205 Host gene (MIR205HG)*, on chromosome 1q32.2. To date, *miR-205* has been widely characterized for its functions in normal development and in cancer, where it was reported to be aberrantly expressed (either up- or downregulated) and exert pro- or anti-tumorigenic roles depending on the cellular context and target genes. Elucidating the mechanisms and factors regulating *miR-205* expression in different biological contexts represents a fundamental step for a better understanding of the contribution of such pivotal miRNA in epithelial cell function and cancer.

This review highlights the recent understanding of *miR-205* transcriptional and post-transcriptional regulation in normal and cancer cells and reveals new insights into the miRNA/host gene architecture. Knowledge in such field may potentially uncover new opportunities to manipulate *miR-205* expression for therapeutic purposes in cancer.

## *miR-205* expression and functions

### *miR-205* in normal physiology

*miR-205* is highly expressed in human epithelial tissues of breast, prostate, skin, eye, and thymus, where it plays a critical role in tissutal morphogenesis and homeostasis. In general, it sustains the epithelial phenotype through the direct targeting of zinc finger E-box-binding homeobox 1 (*ZEB1*) and *ZEB2*, two transcription factors (TFs) that repress *E-cadherin* and other polarity genes^8^.

In the early stages of embryogenesis, *miR-205* is expressed in trophoblasts, where it regulates the placental development through the suppression of Mediator of RNA polymerase II transcription subunit 1 (*MED1*)^9^. During the embryonic development, *miR-205* regulates the extraembryonic endoderm differentiation and spermatogenesis by targeting genes governing cell migration and adhesion^10^.

In the mammary gland, *miR-205* is highly expressed in the basal stem cells. Overexpression of *miR-205* was shown to induce the expansion of the progenitor cell population, reduce cell size and increase cellular proliferation. These effects are mediated by the repression of phosphatase and tensin homolog (*PTEN)* tumor-suppressor^11^. In addition, *miR-205* expression is enriched during gestation and late involution stages, suggesting a dynamic regulation in developmental processes^12^. Similarly, in the human prostate, it is highly expressed in the basal cells, which are presumed as the progenitors of the epithelium. In this context, it regulates the production of the basement membrane protein complex laminin-332 and its receptor integrin-β4, thus ensuring proper tissue polarity and morphogenesis^13^. A *miR-205* involvement in the stemness program has been reported in the skin epidermis and in the stratified epithelia of esophagues and tongue. Here, it plays a pivotal role in the expansion of stem cell population via regulation of PI(3)-kinase signaling^14^. Moreover, by acting on the same signaling pathways, *miR-205* increases the migration of human epidermal and corneal epithelial keratinocytes, playing an essential role in the wound healing and in the corneal development^15^. Consistent with the aforementioned functions, *miR-205* knock-out mice perinatally die due to severe skin defects and compromised proliferation of multiple stratified epithelia tissues^14,16^. In addition, in mice, *miR-205* plays a critical role in the early lacrimal gland development by modulating fibroblast growth factor 10 (FGF10) signaling^17^. Finally, *miR-205* is highly expressed in thymic epithelial cells, where it helps to preserve T-cell maturation in response to strong inflammatory insults, such as infections, radiation exposure, and steroids. This happens through regulation of chemokine/chemokine receptor pathways, antigen processing components, and Wnt signaling system, as a consequence of *miR-205*-mediated repression of *Forkhead Box N1* (*Foxn1*) TF^18^.

Therefore, tight regulation of *miR-205* expression is required for the development of different epithelial tissues. On the other hand, alteration of its normal expression is observed during the initiation and progression of different epithelial cancers.

### *miR-205* in cancer: ups and downs

*miR-205* was found to be either up- or downregulated in several cancers according to the subtype, cell of origin or stage of tumor progression (Table 1). In specific cell types, *miR-205* facilitates tumor initiation and proliferation acting as an oncogene; in others, it inhibits cell proliferation, invasion and epithelial-mesenchimal transition (EMT), thus playing a tumor-suppressive role. Inasmuch as *miR-205* is a marker of epithelial phenotype, its expression was shown to decrease during EMT^8^, a key step in the promotion of tumor invasion and metastasis^11,19^. In accordance to its involvement in this process, the downregulation of *miR-205* has been found in invasive and mesenchymal tumors when comparing to normal tissues^8^. In prostate cancer, *miR-205* was demonstrated to act as a tumor-suppressor by repressing the expression of factors (N-chimerin, E2F1, E2F5, ZEB2, and protein kinase Cε) involved in EMT, cell motility and invasion^20^. Moreover, it was hypothesized to counteract tumor initiation and progression by preserving basement membrane or by repressing the activity of mitogen-activated protein kinase (MAPK) and androgen receptor (AR)^13,21^. Likewise, *miR-205* is downregulated in triple-negative breast cancer (TNBC) and in metastatic breast cancer, indicating a tumor-suppressive role also in these cancer subtypes. Specifically, by targeting human epidermal growth factor receptor 3 (HER3), *miR-205* inhibits cell proliferation and migration and, by repressing *ZEB1* and *ZEB2*, it limits tumor invasion^8,22,23^. Decreased levels of *miR-205* have been reported also in melanoma specimens and in renal cancer, where it exerts anti-proliferative and pro-apoptotic functions by repressing E2F1 and Src-family-genes, respectively^24,25^. On the other hand, *miR-205* is over-expressed in endometrial cancer, where it inhibits apoptosis and promotes cell proliferation through the inhibition of the tumor-suppressors PTEN and estrogen-related receptor-γ (ESRRG)^26,27^. Moreover, in this tumor type, its enhanced levels are related to advanced stage and poor overall survival, suggesting a possible use as prognostic marker^28,29^. Likewise, increased expression of *miR-205* in nasopharyngeal carcinoma is associated with PTEN reduction, followed by tumor promotion and increased resistance to radiotherapy in patients with higher clinical stages^30,31^.

**Table 1:** Summary of the expression, regulation, and functions of *miR-205* in different cancers.

Cancer type	*miR-205* Expression	Regulatory mechanisms	Functions	Target genes	References
Prostate cancer	Downregulated	TFs (p63/ΔNp63α, HIF-1α), hypermetilation, deacetylation	Tumor-suppressor	N-chimerin, E2F1, E2F5, ZEB2, protein kinase Cε, MED1, MAPK, AR	^13,20,21,37,39,40,45,48^
Breast cancer	Downregulated (TNBC)	TF (p53), hypermetilation, deacetylation, lncRNA (*linc-ROR*)	Tumor-suppressor, oncogene	ZEB1, ZEB2, PTEN, HER3, VEGFA, FGF2	^8,11,22,23,38,65^
Lung cancer	Downregulated (adenocarcinoma) or upregulated (squamous cell carcinoma)	Hypermethylation, deacetylation	Tumor-suppressor or oncogene; biomarker	PTEN, PHLPP2, RUNX1	^35,41,51^
Renal cancer	Downregulated	lncRNA (*LINC00152*)	Tumor-suppressor	Src-family members, Ras/Raf/ERK1/2	^25,66^
Head and neck cancer	Upregulated	TF (p53)	Oncogene, prognostic marker	PTEN	^30^
Melanoma	Downregulated	TFs (p73/ΔNp73)	Tumor-suppressor	E2F1, BCL2, VEGF	^24^
Bladder cancer	Downregulated invasive bladder cancer) or upregulated (non-invasive bladder cancer)	TF (ΔNp63α, Twist1), hypermetilation, lncRNA (*HOTAIR*)	Tumor-suppressor or oncogene	ZEB1, ZEB2	^36,47,67^
Endometrial cancer	Upregulated	lncRNAs (*RP11–395G23.3* and *LA16c-313D11.11*)	Oncogene, prognostic marker	PTEN, ESRRG	^26,27^

In addition, *miR-205* levels were significantly increased in the serum of ovarian cancer patients and high expression of the circulating *miR-205* was associated with angiogenesis and ovarian cancer progression^32,33^. In this context, the reduction of the *miR-205* PTEN target leads to a persistent activation of AKT signaling, which results in uncontrolled proliferation and neoplastic angiogenesis^33^.

Accumulating evidence showed heterogeneity of *miR-205* expression within the same tumor type, as reported in non-small cell lung carcinoma and esophageal cancer. In these contexts, *miR-205* overexpression is indicative of squamous cell carcinoma, while its down-expression is characteristic of adenocarcinoma, thus suggesting its possible application as a diagnostic marker^34,35^. Taken together, these studies provide evidence for the dual role of *miR-205* in different cancers and arise important questions about upstream factors regulating its expression.

## Levels of *miR-205* regulation

### Transcriptional regulation: the players and the molecular landscape

#### Chromatin changes

Epigenetic modifications, especially Cytosine (phosphodiester bond) Guanine (CpG) DNA methylation and histone re-modeling, have important roles in gene expression regulation and can also affect miRNA transcription. The recruitment of DNA methyltransferase (DNMT) and histone deacetylases (HDACs) leads to chromatin inactivation and the consequent repression of transcription. CpG sites were identified in a region immediately upstream of the first exon of *MIR205HG* and in the *miR-205* locus^22,36–38^. For example, DNA methylation in the 300 bases preceding *MIR205HG* Transcription Start Site (TSS) and deacetylation of lysine 9 of histone 3 (H3K9) in the genomic region coding for pre-*miR-205* were shown to contribute to *miR-205* downregulation in prostate cancer cells^37,39^. Other evidence for the *miR-205* repressive epigenetic status in prostate cancer cells has been reported by Ke and colleagues, who detected a gain of lysine 27 trimethylation of histone 3 (H3K27me3) mark and a loss of lysine 4 trimethylation of histone 3 (H3K4me3) in *miR-205* locus^40^. Similarly, aberrant DNA hypermethylation, H3K9 deacetylation and H3K27me3 in the pre-*miR-205* locus are related to *miR-205* silencing in muscle-invasive bladder cancer and in transformed lung epithelial cells^36,41^. However, treatment with de-methylating agents and histone deacetylase (HDAC) inhibitors demonstrated that these chromatin modifications are not the sole regulators of *miR-205* and that other factors may contribute to its transcriptional repression^36^. In another aggressive subtype of breast cancer, defined as HER2-positive cancer, *miR-205* expression is modulated by HER2 signaling via Ras/Raf/MEK/ERK. The activation of this pathway induces the overexpression of DNMT proteins and the consequent hypermethylation of the regions upstream of *MIR205HG* and of *miR-205* locus^38^. On the contrary, DNA hypomethylation was responsible for the induced *miR-205* expression in oral squamous cell and ovarian carcinomas^32,42^. Indirectly, the epigenetic status can regulate the expression of *miR-205* by the modulation of TFs. As reported in TNBC, the restoration of the normal DNA methylation status induces p53 TF up-regulation and the consequent rebalancing of *miR-205* levels^22^.

#### Transcription factors

*miR-205* expression was shown to be regulated by several TFs, among which the members of p53 family, Specificity protein 1 (Sp1), Hypoxia-Inducible Factor alpha (HIF-1α), Transforming Growth Factor β (TGF-β), and Twist-related protein 1 (Twist1)^13,22,36,43–48^.

**Fig. 1:**
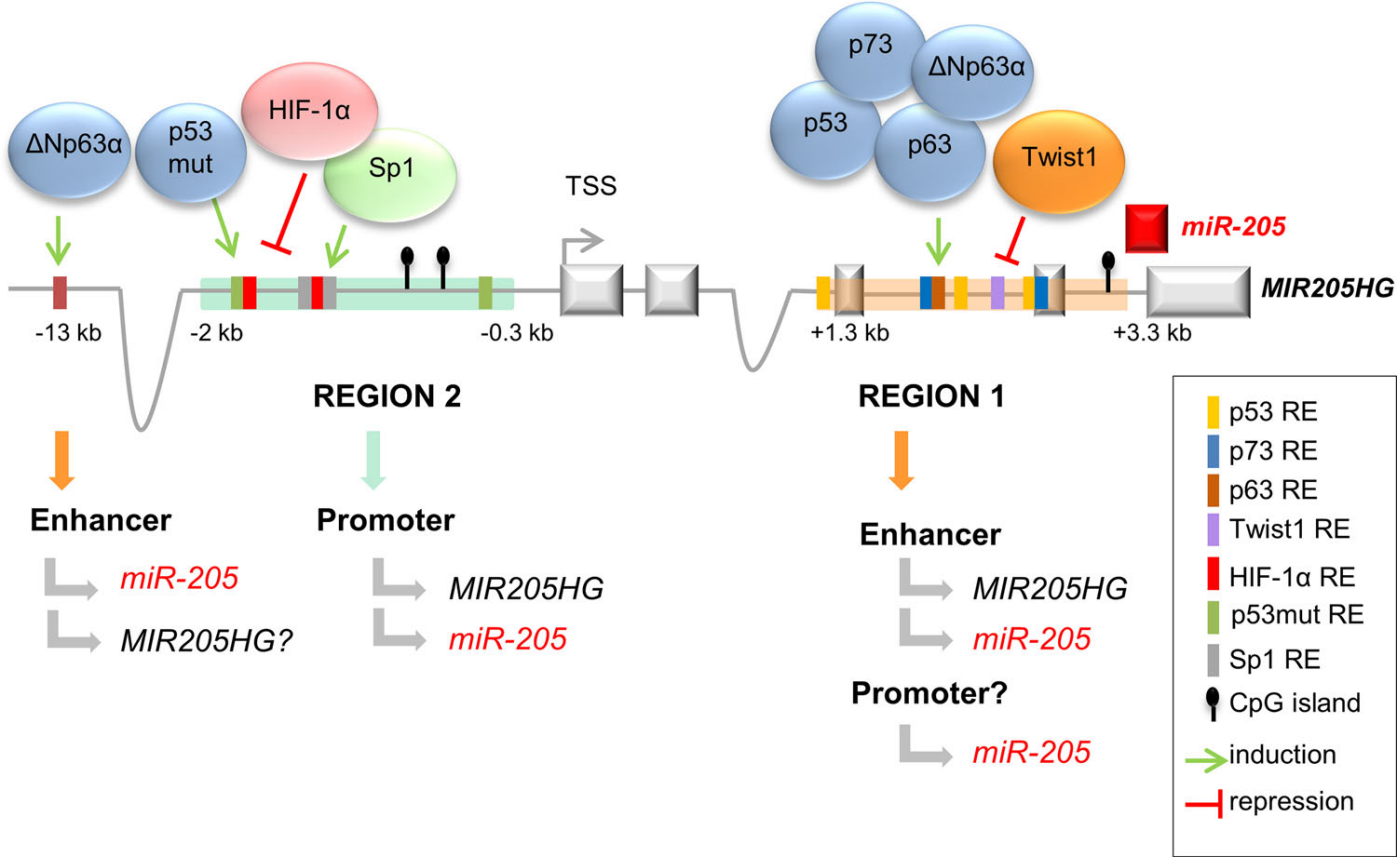
Human *miR-205* host gene (*MIR205HG*) transcription unit. The schematic representation shows the transcription factor responsive elements (REs) and methylation sites (CpG islands) localized upstream of *MIR205HG* and of pre-*miR-205* sequence. The *miR-205* and *MIR205HG* regulatory regions are depicted in orange (region 1) and in aquamarine (region 2).

P53 plays multiple roles, including regulation of the cell cycle, apoptosis and genomic stability, thus exerting a tumor-suppressive function^49,50^. Findings showed that p53 stimulates the expression of *miR-205* by interacting with putative p53 responsive elements (p53REs) in a region upstream of *miR-205* sequence, in different cellular models^22^ (Fig. 1). Notably, a decrease or complete loss of *miR-205* is frequently found in breast cancers. In this regard, it was reported that inactivating mutations of p53 that result in its defective binding to p53REs are responsible for decreased level of *miR-205* in TNBC^22^. Conversely, p53 gain of function mutants (GOF-mutp53) induce the up-regulation of *miR-205*, which promotes proliferation in head and neck squamous cell carcinoma. Interestingly, GOF-mutp53 does not recognize the wild type-p53RE in the pre-*miR-205* genomic sequence but binds to a region upstream of *MIR205HG*^51^. p53 mutants may impinge on *miR-205* expression also through different mechanisms. For example, in the prostate cancer context, p53 mutants reduce p63 stability and activity, with a consequent reduction of *miR-205* transcriptional rate^48^. In fact, p63 and p73 proteins, by exhibiting a remarkable sequence and structural homology to p53, can bind to p53 DNA target sites and similarly regulate *miR-205* expression^24,52^. In prostate and bladder epithelia, p63 and its isoform ΔNp63α, lacking the transactivation domain, bind to p53REs upstream of *miR-205* sequence and induce its expression^13,47,48^. As consequence, the loss of p63 observed in these epithelia upon transformation results in a decrease of *miR-205* levels and in a reciprocal effect on EMT process^47,48^. On the other hand, p73 TF, by binding to *miR-205* upstream sites, induces *miR-205* expression in non-metastatic melanoma cells. Interestingly, the truncated form of p73, ΔNp73, highly expressed in malignant melanoma cells, interferes with p73 activity, thus being responsible for the reduced expression of *miR-205*^24^.

Another DNA-binding factor that regulates *miR-205* expression is Sp1. It was shown that induction of Sp1 upon DNA damage activates the expression of *miR-205* and its host gene in esophageal squamous cell carcinoma by binding to sites immediately upstream of *MIR205HG*. Interestingly, the Sp1-mediated transcriptional activation of *miR-205* promotes radioresistence and an aggressive phenotype through PTEN-PI3K/AKT pathway^46^. Conversely, pro-inflammatory cytokines, cell or environmental stresses converge onto the downregulation of *miR-205* in EMT contexts. A decreased expression of *miR-205* was reported in a canine kidney (MDCK) and in a glioma cell lines upon treatment with TGF-β1^8,44^. Moreover, TGF-β1, co-secreted with IL-6 by aggressive prostate cancer cells, converts stromal cells into cancer-associated fibroblasts, which in turn stabilize HIF-1α. This redox sensitive TF, directly interacting with REs in proximity of *MIR205HG*, represses *miR-205* expression. In this context, downregulation of *miR-205* leads to de-repression of *ZEB1/2* and *PKCε*, allowing EMT of prostate cancer cells^45^. Again, *miR-205* expression was shown to be controlled by the EMT-inducing transcription Twist1. Indeed, Twist1 was proven to directly repress *miR-205* transcription in invasive bladder cancer, through direct binding to a pre-*miR-205* regulatory region^36^.

#### *miR-205* transcription unit: insights into the regulatory regions

Pre-*miR-205* sequence is located within *MIR205HG*, precisely in the connecting region between its last intron and exon. This peculiar location stimulated interest in investigating the potential transcriptional relationship between the two genes and in identifying the relevant regulatory regions. As mentioned before, TF motifs, DNA methylation and DNAseI-sensitive sites were found in proximity of both pre-*miR-205* (region 1, Fig. 1) and *MIR205HG* (region 2, Fig. 1), suggesting a possible regulatory role for these regions. Region 1 is located within 2 kb upstream of the pre-*miR-205* sequence and contains p53 family REs, Twist1-binding sites and methylation sites^13,22,24,47,48^. Region 2 is upstream of the first exon of *MIR205HG* and includes binding sites for GOF-mutp53, HIF-1α, Sp1, and chromatin modification sites^37–39,43,45,46^. In addition, a sequence at –13 kb with respect to the *MIR205HG* TSS, enriched in p63 sites, is functional in regulating *miR-205* expression^13^. A number of research groups investigated the regulatory role of these regions through in silico and experimental analyses. Region 1 showed to have promoter activity in two different works, when tested by reporter assay upon co-transfection with p63 and p73^24,48^. However, the same region tested by a different reporter construct showed only enhancer activity upon p53 transfection in HEK-293 cells^22^. In addition, Chromatin immunoprecipitation (ChIP) experiments demonstrated that the functional binding of the p63 isoform ΔNp63 to responsive elements in region 1 induced a strong enrichment of RNA Polymerase II onto region 2, thus resulting in increased expression of both *miR-205* and *MIR205HG* transcripts^47^. Consistent with this, region 2 was validated to have promoter activity in reporter assays by different authors^22,45,46^. The presence of CpG in region 2 again supports its promoter function, as methylation sites in human genes are mostly related to promoter regions^37–39,53^. A recent work showed that the binding of the mutant p53 to region 2 increases the expression of both *MIR205HG* and *miR-205*^43^, indicating that this region may act as the promoter for both RNAs. Consistent with this, analysis of RNA-seq data from The Cancer Genome Atlas (TCGA, https://www.cancer.gov/tcga) showed marked correlation between *MIR205HG* and *miR-205* expression across tissues^54^, again supporting co-transcriptional regulation. Recent data from our lab showed that genomic deletion of the sequence spanning from exon 1 to 3 of *MIR205HG* (including TSS) in prostate basal cells or antisense oligonucleotide-mediated targeting of introns of *MIR205HG* primary sequence invariably resulted in the abrogation of both *MIR205HG* and *miR-205* expression^54^, letting to hypothesize that a “promiscuous” *MIR205HG*/*miR-205* primary transcript might exist (see section “Post-transcriptional regulation” for further details).

Altogether, the evidence collected so far suggests two main possible models for *miR-205* transcription: the first one accounts for an independent *miR-205* transcription driven by pre-*miR-205* proximal promoter (region 1, Fig. 1); the latter supports a *MIR205HG*-dependent expression dictated by the distal promoter (region 2, Fig. 1), which would transcribe for a common *MIR205HG*/*miR-205* primary transcript, with region 1 acting as enhancer.

### Post-transcriptional regulation

#### *miR-205* processing: implications for *miR-205* and *MIR205HG* expression

**Fig. 2:**
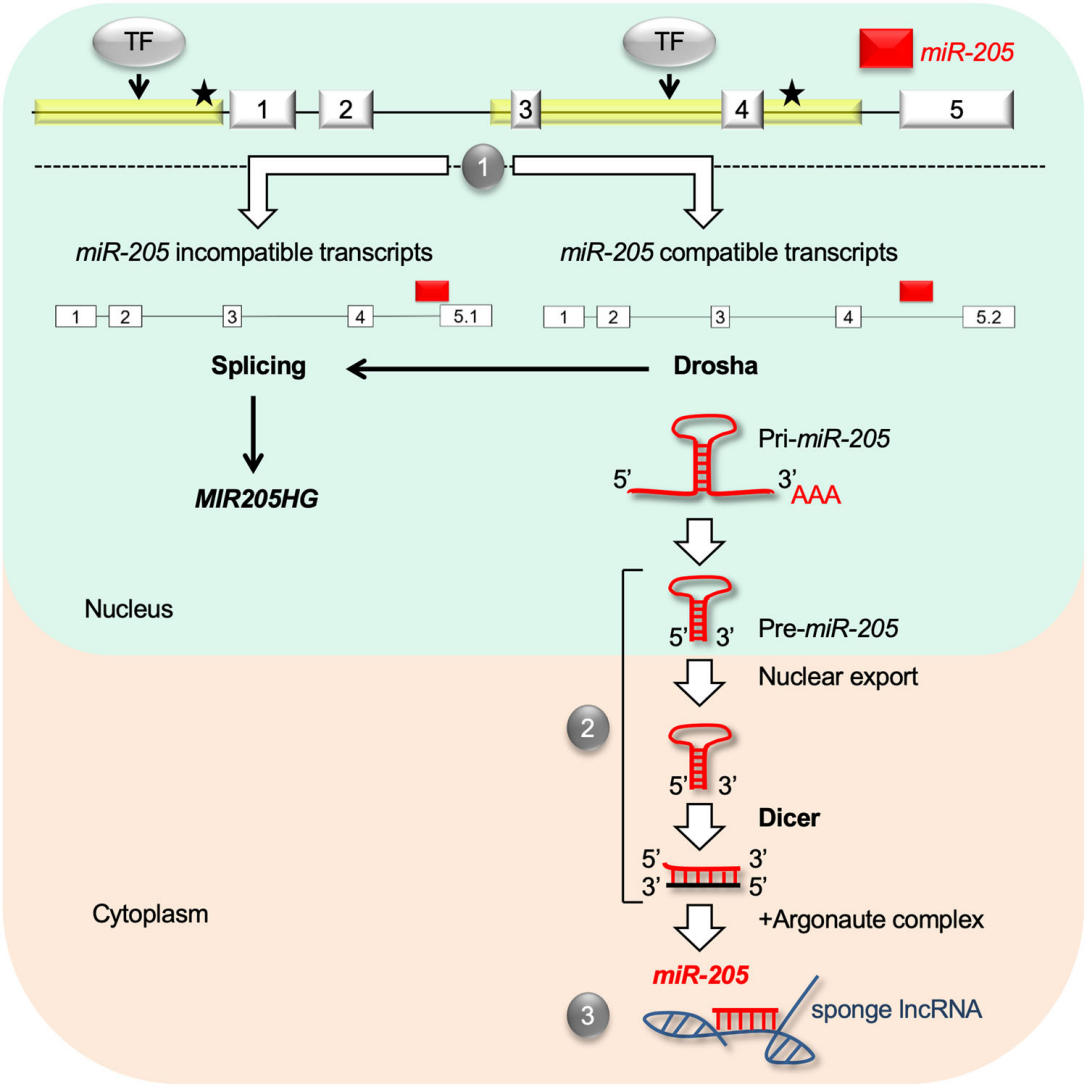
Scheme of *miR-205* regulation. At the transcriptional level, *miR-205* is regulated by transcription factors (TF) or epigenetic modifications (indicated by stars). At the post-transcriptional level, alternative splicing of *MIR205HG* primary transcript gives origin to *miR-205* compatible and non-compatible transcripts (1). Drosha masks the splicing site between *MIR205HG* exons 4 and 5.1, favoring the use of an alternative exon (exon 5.2) and the excision of pre-*miR-205*, which is then processed by Dicer into miRNA duplex (2). *miR-205* bioavailability is regulated by several cytoplasmic lncRNAs acting as miRNA sponges (3).

The peculiar pre-*miR-205* location in the last intron-exon junction of *MIR205HG* arises important questions about *miR-205* biogenesis from the putative *MIR205HG*/*miR-205* primary transcript. In fact, use of this splice site would be incompatible with the excision of pre-*miR-205*. Interestingly, Chang and co-workers^55^, by sequencing pri-miRNA structures using a dominant-negative Drosha mutant, were able to map lowly expressed alternatively spliced *MIR205HG* transcripts that, by utilizing a distinct 3’ terminal exon (exon 5.2, Fig. 2), make the pre-*miR-205* sequence fully intronic, a configuration permissive to miRNA processing. Accordingly, reannotation of all possible *MIR205HG* transcripts made starting from recently acquired long read sequencing data^56^ suggested the existence of two locus configurations, one that acts as source of *miR-205* (*miR-205* compatible transcripts) and the other that gives rise to *MIR205HG* transcripts only (*miR-205* incompatible transcripts)^54^. These data suggest that alternative splicing may dictate the switch between the two locus configurations.

Notably, the process of *miR-205* production from *miR-205* compatible transcripts was found to be dependent on Drosha activity. Specifically, Drosha is not only responsible for miRNA processing, rather it is able to physically mask *miR-205*-incompatible splicing site, thus favoring production of *miR-205*-compatible *MIR205HG* isoforms^54^. Consistent with this, defects in biogenesis machinery were observed in prostate cancer cells under hypoxia where the decreased levels of Drosha and Dicer resulted in a down-modulation of *miR-205*^45,57^. Taken together, these findings illustrate an additional level of *miR-205* regulation where competition between the spliceosome and the microprocessor Drosha may direct the processing of *MIR205HG* primary transcript into *miR-205* and/or *MIR205HG* RNAs. In this regard, it is still to explore whether a functional splicing machinery is requested or dispensable for *miR-205* biogenesis.

It is worth mentioning that, though processed from a common primary transcript, mature *MIR205HG* and *miR-205* have been shown to act independently. In fact, recent studies on *MIR205HG* support its function as long noncoding RNA (lncRNA) in different cell contexts (Table 2)^43,54,58–62^. LncRNAs are functional non-protein coding transcripts longer than 200 nucleotides^63^. In the last years, lncRNAs have been increasingly recognized as regulators of pivotal biological processes, with their aberrant activity being implicated in tumorigenesis^64^.

**Table 2:** Summary of the reported functions and mechanisms of action of *MIR205HG* lncRNA in different tissue/tumor types.

Species	Tissue/cancer type	Biological role	Mechanism of action	References
Human	LUSC	Cell proliferation, migration, EMT	ceRNA for *miR-299–3p*	^62^
Human	LUSC	Cell proliferation, migration, apoptosis	Bcl-2 and Bax regulation	^61^
Human	Cervical cancer	Cell proliferation, migration, apoptosis	SRSF1/KRT17 axis regulation	^60^
Human	Cervical cancer	Cell proliferation, migration, apoptosis	ceRNA for *miR-122-5p*	^59^
Human	HNSCC	Cell proliferation, migration	ceRNA for *miR-590-3p*	^43^
Human	Prostate basal cells	Basal-luminal differentiation	transcriptional repression of interferon genes	^54^
Mouse	Pituitary	Growth hormone and prolactin production	*Pit1* regulation	^58^

*LUSC* lung squamous cell carcinoma, *HNSCC* head and neck squamous cell carcinoma.

In head and neck squamous cell carcinoma, where both *MIR205HG* and *miR-205* are over-expressed, *MIR205HG* lncRNA was shown to enhance cancer cell proliferation and motility by sequestering *miR-590–3p* and preventing interaction with its target mRNAs, thus acting as “molecular sponge”^43^. Similar protumorigenic effects were reported for *MIR205HG* also in cervical cancer^59,60^ and in lung squamous cell carcinoma^61,62^, though with different mechanisms of action (Table 2). This oncogenic role is remindful, though independent, to that of *miR-205* in squamous cell carcinomas, where the miRNAs was shown to exert protumorigenic function through repression of tumor-suppressor genes^46^. *MIR205HG* and *miR-205* were reported to play non redundant independent functions also in prostate basal cells, where *MIR205HG* maintains basal identity by regulating differentiation and *miR-205* regulates the production of basement membrane^13,54^. Notably, also in the mouse context, *MIR205HG* lncRNA was shown to work independently of *miR-205* in regulating growth hormone and prolactin production in the anterior pituitary^58^.

#### Reduction of *miR-205* availability: role of long noncoding RNAs

One of the numerous mechanisms by which lncRNAs can regulate gene expression is by sponging (sequestering) miRNAs. Owing to their frequent aberrant expression in cancer, they are in part responsible for the dysregulated miRNA expression in such conditions^65,66^. Growing evidence reported a negative control relationship between *miR-205* and lncRNAs in cancer. The long Intergenic Non-Protein Coding 00673 (*LINC00673)*, enriched in hepatocellular carcinoma, by adsorbing *miR-205* reduces its availability and consequently prevents *miR-205* tumor-suppressor functions^67^. Small Nucleolar RNA Host Gene 5 (*SNHG5*) is another tumor-enriched lncRNA that binds and sequesters *miR-205*, de-repressing the target ATP-binding cassette sub-family C member 2 (ABCC2) and promoting imatinib resistance in chronic myeloid leukemia^68^. On the other hand, reduced levels of the lncRNA growth arrest specific 5 (*GAS5*) in human cervical cancer dictate the up-regulation of *miR-205* and its oncogenic role in promoting the proliferation and migration of cervical cells^69^. A crosstalk between lncRNAs and *miR-205* has been also observed in renal carcinoma, where a reduction of *miR-205* is related to an overexpression of *LINC00152*^70^. *RP11–395G23.3* and *LA16c-313D11.11* are two lncRNAs associated to the pathogenesis of endometrial cancer. They act as endogenous competing RNAs for *miR-205*-PTEN network and have been shown to inhibit the expression and the activity of *miR-205* at the post-transcriptional level through highly conserved miRNA responsive elements^71^. Again, a recent work reported a link between decreased levels of *miR-205* and high expression of lncRNA small nucleolar RNA host gene 16 (*SNHG16*) in osteosarcoma tissues. These studies revealed that *SNHG16*, by acting as endogenous sponge of *miR-205*, upregulates ZEB1 and enhances proliferation of osteosarcoma cells^72^. Similarly, *SNHG16* was found to act as *miR-205* sponge in the cardiovascular context, where reduced levels o*f miR-205* are related to increased proliferation and migration of aortic smooth muscle cells, suggesting a link with atherosclerosis^73^. In ovarian cancer, an increase of *miR-205* levels are related to a reduction of its lncRNA sponge *LINC01133*. This unbalance results in a reduction of the *miR-205* target Leucine-rich repeat kinase 2 (LRRK2) and enhanced proliferatory, migratory and invasive ovarian cancer cell ability^74^.

The *LINC* regulator of reprogramming (*LINC-ROR*) is important for the maintenance of induced pluripotent and embryonic stem cells. Specifically, *LINC-ROR*, by acting as a molecular sponge for *miR-205*, prevents the degradation of *miR-205* targets (e.g., *ZEB1* and *ZEB2*) and promotes EMT in breast cancer^75^.

Interestingly, lncRNAs can also indirectly regulate miRNAs. As reported by Sun and co-workers^76^, the lncRNA HOX transcript antisense RNA (*HOTAIR*) participates to *miR-205* silencing in bladder cancer by breaking the balance between the positive (H3K4me3) and negative (H3K27me3) chromatin marks on *miR-205* promoter.

## Conclusions

*miR-205* is one of the most investigated miRNAs due to its involvement in multiple physiologic, oncogenic and tumor-suppressor pathways. Several mechanisms and factors, depending on cell and tumor types, regulate *miR-205* expression and contribute to its complex function. In cancer, epigenetic modifications, mutated or alternatively spliced p53 family proteins and components of tumor microenvironment (hypoxia, inflammatory cytokines) mostly contribute to *miR-205* dysregulation at the transcriptional level. Post-transcriptionally, lncRNAs are in part responsible for changes in *miR-205* availability in tumor cells.

*miR-205* regulation is made further complicated by its special genomic location, which rises issues regarding its biogenesis in relationship to that of its host gene. Despite the binding of transcription factors upstream of the precursor *miR-205* sequence could account for a *MIR205HG*-independent transcription, experimental data supporting promoter activity of this region are weak. The most accredited function for this regulatory region could be to serve as enhancer for transcription of a common *MIR205HG*/*miR-205* primary transcript, from which both *miR-205* and *MIR205HG*-processed transcripts are produced. However, additional investigations in the sequences upstream or downstream of the pre-*miR-205* could evidence regions involved in specific *miR-205* regulation.

The observation that *miR-205* can originate from alternatively spliced *MIR205HG* transcripts with the intervention of Drosha lets to speculate that a diverse commitment between *miR-205* compatible and incompatible transcripts and/or a competition between Drosha and spliceosome may represent a further checkpoint in *miR-205* regulation to modify *miR-205*/*MIR205HG* ratio. The fine tuning between *miR-205* and *MIR205HG* level may have important implications on biological functions, in dependence on the cell type and physio-pathological state. In this regard, additional investigation would be required to identify the context and factors that may modify reciprocal *miR-205*/*MIR205HG* biogenesis.

Overall, the reviewed studies provide a further understanding of *miR-205* gene organization and regulation, including the recently acquired knowledge about its host gene. Elucidating the complex, multilevel regulation of *miR-205* expression during the normal development of epithelia is crucial to determine which factors maintain the balance and contribute to the physiologic state. Subsequently, unraveling the causes responsible for *miR-205* dysregulation in cancer is of utmost interest for the future development of *miR-205* modulating strategies applicable in therapy.
